# The viral etiology of EBV-associated gastric cancers contributes to their unique pathology, clinical outcomes, treatment responses and immune landscape

**DOI:** 10.3389/fimmu.2024.1358511

**Published:** 2024-03-26

**Authors:** Mikhail Y. Salnikov, Katelyn M. MacNeil, Joe S. Mymryk

**Affiliations:** ^1^ Department of Microbiology and Immunology, Western University, London, ON, Canada; ^2^ Department of Oncology, Western University, London, ON, Canada; ^3^ Department of Otolaryngology, Western University, London, ON, Canada; ^4^ Lawson Health Research Institute, London, ON, Canada

**Keywords:** Epstein-Barr virus, gastric cancer, EBVaGC, tumor microenvironment, immune landscape, therapeutics, tumor virus, immunotherapy

## Abstract

Epstein-Barr virus (EBV) is a pathogen known to cause a number of malignancies, often taking years for them to develop after primary infection. EBV-associated gastric cancer (EBVaGC) is one such malignancy, and is an immunologically, molecularly and pathologically distinct entity from EBV-negative gastric cancer (EBVnGC). In comparison with EBVnGCs, EBVaGCs overexpress a number of immune regulatory genes to help form an immunosuppressive tumor microenvironment (TME), have improved prognosis, and overall have an “immune-hot” phenotype. This review provides an overview of the histopathology, clinical features and clinical outcomes of EBVaGCs. We also summarize the differences between the TMEs of EBVaGCs and EBVnGCs, which includes significant differences in cell composition and immune infiltration. A list of available EBVaGC and EBVnGC gene expression datasets and computational tools are also provided within this review. Finally, an overview is provided of the various chemo- and immuno-therapeutics available in treating gastric cancers (GCs), with a focus on EBVaGCs.

## 1 Introduction

Epstein-Barr virus (EBV) is a gamma-herpesvirus most known for influencing B lymphocyte proliferation and differentiation to a plasmablast/early plasma cell-like phenotype (1, 2), as well as causing cytopathic effects within mucosal epithelial cells (3). More rarely, EBV infects T and NK cells, causing lymphoproliferative disorders (4). By evading both the innate and adaptive immune systems, EBV can establish lifelong, latent infections within B lymphocytes, which can lead to reactivation and further infections (5). In fact, EBV is so successful that it is estimated that over 90% of the world population may be infected with it (6).

EBV is also associated with a number of different cancers, which include nasopharyngeal carcinomas (NPCs), EBV-associated gastric cancers (EBVaGCs), as well as Burkitt and other lymphomas (7, 8). Overall, EBV infections account for 1.5% of all cancers globally (9), with 84.6% (10), 8.8% (11), and 50% (10) of NPCs, gastric cancers (GCs), and Hodgkin’s lymphomas attributable to EBV infections, respectively. Currently, there are no approved vaccines against EBV, but there are a number of prophylactic and therapeutic vaccines currently undergoing trials (12–14). However, there are several approved therapies targeting EBV-associated cancers with various levels of effectiveness (9, 15).

Gastric cancer is the fifth most diagnosed cancer, with over a million new cases annually, and is the third most common cause of cancer-related death (16). GCs have a number of risk factors, which include *Helicobacter pylori (H. pylori)* and EBV infections, genetic and dietary factors, as well as smoking status and alcohol consumption (17–19). The etiology of EBV in GCs was first identified in 1990 by Burke et al. (20), with Shibata and Weiss demonstrating the presence of the EBV genome within cancerous and dysplastic cells (21). EBVaGCs have been established as molecularly and clinically distinct from EBV-negative GCs (EBVnGCs) by The Cancer Genome Atlas (TCGA), which defined 4 different subtypes of EBVnGCs: microsatellite-instable (MSI) tumors, genomically stable (GS) tumors, tumors with chromosomal instability (CIN), and tumors with DNA polymerase epsilon mutations (POLE) (22).

## 2 EBV infection of gastric epithelial cells

**Figure 1:**
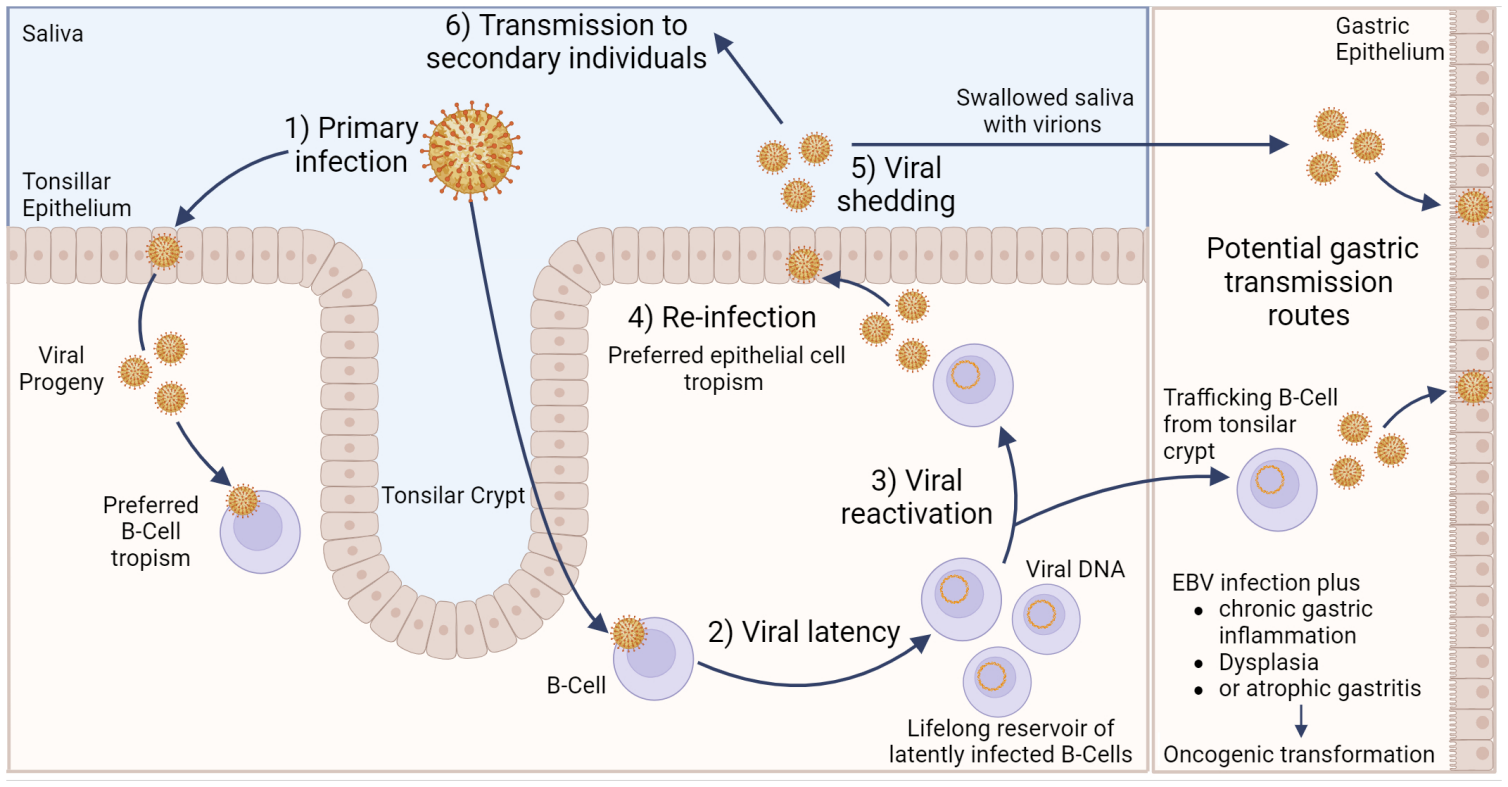
Model of Epstein–Barr virus infection in relation to gastric cancer. EBV can infect tonsillar epithelium or B cells located in the tonsillar crypt, with the resulting viral progeny having a preferred tropism for the opposite cell type. Following B cell infection, EBV can establish life-long latency in pools of B cells. Occasionally, these cells can become reactivated, resulting in subsequent release of viral progeny that exhibit a preferred epithelial cell tropism. Viral shedding from epithelial cells into salvia can result in EBV transmission to secondary individuals. Alternatively, salvia containing viral progeny can be swallowed, resulting in infection of gastric epithelial cells. Likewise, latently infected, trafficking B cells can undergo reactivation, and the resulting viral progeny can then infect gastric epithelial cells. Either of these potential routes could lead to gastric epithelial cell infection, which may subsequently lead to EBVaGC. Created with BioRender.com.

EBV transmission between individuals is typically mediated by saliva (23). Indeed, EBV causes infectious mononucleosis, which is sometimes referred to as the “kissing disease”. Primary infection of a naïve individual typically occurs when incoming EBV virions directly infect susceptible B cells in the tonsillar crypt (**Figure 1**). Alternatively, an incoming virus may directly infect tonsillar epithelial cells, which produce progeny viruses that later infect B cells present in the crypts. EBV establishes latency in the infected B cells, creating a reservoir of virus-infected B cells that persist throughout the life of the infected individual. Latently infected B cells occasionally undergo reactivation, producing viruses that reinfects oral epithelial cells. These cells then shed viruses into the saliva at high titre, allowing transmission to naïve individuals periodically throughout the lifetime of the infected individual (23). Interestingly, viruses released from infected B cells preferentially infect epithelial cells, while viruses released from infected epithelial cells preferentially infect B cells. The molecular basis for this switch in tropism is related to changes in the glycoprotein composition in the envelopes of B cell-derived and epithelial cell-derived viruses and helps to reinforce alternate replication between the two cell types (24).

Direct infection of gastric epithelial cells could occur from swallowing saliva containing EBV virions shed from their own infected oral epithelium (**Figure 1**). This process would be akin to virus transmission from person to person, as described above, except that the infection is spreading from one anatomical location to another in the same individual. Alternatively, a productive EBV infection could be reactivated in latently infected B cells trafficking through the gastric mucosa and released to infect neighbouring epithelial cells (24). In support of this route of infection, coculture of epithelial cells with EBV-positive lymphocyte cells is approximately 800-fold more efficient than cell-free infection, suggesting the possibility of direct cell-to-cell mediated virus infection (25). A definitive answer to the exact mechanism by which gastric epithelial cells become infected by EBV remains to be determined.

## 3 EBVaGC histopathology and clinical features

EBVaGCs are considered to be molecularly and pathologically distinct entities from EBVnGCs (9, 26–28). This section will highlight a number of known differences, both macroscopic and microscopic, molecular and morphological, as well as differences related to clinical features and patient outcomes between EBVaGCs and EBVnGCs.

### 3.1 Histomorphology and histopathology

EBVaGCs preferentially develop in the proximal region of the stomach, which includes the cardia, fundus, and body (29), with lymphoepithelioma-like carcinoma and Crohn’s disease-like lymphocytic reaction being the dominant histological subtypes (30). In fact, over 90% of lymphoepithelioma-like carcinomas are EBV-positive (31), where tumor cells are outnumbered by tumor-infiltration lymphocytes (TILs) (29). In contrast, EBVnGCs predominate within the antrum region of the stomach (29) and exhibit a lower infiltration of TILs (32). Gastritis cystica profunda, a precancerous lesion associated with increased proliferation activity and cystic gastric glands within the submucosa, is also frequently associated with a positive EBV-status (33). Immunophenotyping of EBVaGCs indicates an even split between a gastric-like phenotype, expressing both the MUC5AC and MUC6 mucins, and a null phenotype, expressing neither gastric-like, nor intestinal-like phenotypes (34). A detailed description of the histology and immunophenotype of gastric differentiation markers in EBVaGC has been summarized previously (34).

Even though there is a disparity of TILs between EBVaGCs and EBVnGCs, increased infiltration by lymphocytes can lead to the formation of tertiary lymphoid structures in both types of GCs, which often develop their own germinal centers and correlates with CD4+ and CD8+ T cell infiltration (35, 36). Tertiary lymphoid structures are generally associated with better outcomes for most types of cancer (35), including GCs, where the presence of these structures is associated with higher levels of TILs and increased overall survival (32). Further exploration of the histomorphology and histopathology, in conjunction with the immune landscape, of EBVaGCs and EBVnGCs may yield deeper insights into the differences in tumor initiation and progression, as well as structural feature changes associated with these carcinomas.

### 3.2 Identification, clinical features and general outcomes

EBVaGC is commonly identified by *in situ* hybridization with a probe that specifically anneals to the small non-coding EBER1 viral RNA, which is abundantly expressed in EBV infected and transformed cells (37). These probes enable accurate detection of EBV-infected cells with high specificity in formaldehyde-fixed and paraffin-embedded GC samples (38). This is especially relevant, as EBV infections are associated with an 18-fold increased risk of developing GC (11).

Both EBVaGCs and EBVnGCs have a higher incidence in men as compared to women (39, 40). In contrast, EBVaGCs have a better prognosis and greater median survival time (30, 41, 42), an increased presence of TILs (43, 44), increased promotor hypermethylation (45, 46), and higher levels of MHC-I and MHC-II expression (47, 48) compared to EBVnGCs. EBVaGCs are also found more often among young individuals as compared to EBVnGCs (49, 50). A lower density of TILs is also associated with increased presence of lymph node metastasis, an important negative determinant of disease progression in GCs (51) that is also associated with poorer patient outcomes (28). Additionally, co-infections of *H. pylori* and EBV may result in earlier and more aggressive GC progression (52).

### 3.3 Cell markers associated with patient outcomes in EBVaGC

There are a number of differentially expressed cellular genes associated with differences in outcomes for patients with EBVaGC. Programmed death-ligand 1 (PD-L1), an immune checkpoint protein that binds to its receptor PD-1 on T cells and other immune cells, is overexpressed in EBVaGCs. Indeed, in a recent meta-analysis of 43 publications encompassing a total of 11,327 patients, there was a very clear increase in the association between PD-L1 expression and EBVaGC (OR = 6.36, 95% CI 3.91-10.3, p < 0.001) (53). This increase in PD-L1 likely occurs as a response to higher intratumoral levels of IFN-γ via activation of IRF3 and is associated with worse patient outcomes (54, 55). Poorer patient outcomes have also been associated with increased expression of indoleamine 2,3-dioxygenase 1 (IDO1), another potent immune suppressor (56, 57). Human epidermal growth factor receptor 2 (HER2) is a proto-oncogene that is downregulated by LMP2A, an EBV-encoded protein, in some cases of EBVaGC (49, 58). However, the increased expression of HER2 is associated with lower overall patient survival in EBVaGC (59). The identification of additional prognostic markers for EBVaGC will help develop a greater diversity of personalized and targeted therapies (60, 61).

### 3.4 Impact of H. pylori infection on EBVaGC

In addition to EBV, chronic inflammation and tissue damage induced by *H. pylori* infection increases the risk of developing GC (62). Multiple studies suggest that in the context of co-infections, these microorganisms may cooperate to promote infection, inflammation and possibly carcinogenesis (63–66). The mechanisms controlling the interactions between these two infectious agents are not entirely known and have been reviewed in detail by others (67). However, in a recent large cohort study, EBV and *H. pylori* co-infection was not identified as a clinically significant independent prognostic factor for the development of gastric cancer (68). Similarly, EBV and *H. pylori* co-infection did not significantly affect overall survival rate compared to those with EBV alone (68). It should also be noted that co-infection is clearly not essential for carcinogenesis, as EBVaGCs can still occur many years after successful eradication of *H. pylori* (69) and only a fraction of EBVaGCs are co-infected with *H. pylori* (22, 52).

## 4 Role of EBV-encoded proteins in EBVaGC tumor progression

EBV-encoded proteins play a major role in shaping the immune microenvironment of EBVaGCs and promoting tumor growth. EBV encoded latency protein Epstein–Barr nuclear antigen 1 (EBNA1) is uniformly expressed in EBVaGCs, with variable expression of latent membrane protein 1 (LMP1) and latent membrane protein 2A (LMP2A) (70, 71).

### 4.1 The functions of EBNA1 in EBVaGC

EBNA1 is expressed in all forms of EBV latency in proliferating cells and in all EBV-associated tumours (72). It performs critical roles in maintaining the persistence of latent EBV genomes in the nucleus as episomes and also plays a role in transcriptional activation of viral genes (73). Additionally, mounting evidence suggests that EBNA1 functions more directly to impact cell survival and oncogenesis (72). These include antagonism of the Tumor protein P53 (TP53) pathway (74), degradation of promyelocytic leukemia (PML) tumor suppressor protein (75), modulation of various signal transduction pathways (76), and increased oxidative stress (77). Most recently, a previously unknown link between EBV and genomic instability between EBNA1-induced breakage at 11q23 and the acquisition of chromosome 11 structural variations was identified, but this has yet to be specifically confirmed in EBVaGC (78).

Interestingly, specific amino acid changes in EBNA1 have been identified that were strongly associated with viruses isolated from EBVaGCs and NPCs, but not isolates from lymphoma and healthy individuals (71). One of these mutations (Thr85Ala) results in a gain of function interaction with the procollagen-lysine,2-oxoglutarate 5-dioxygenase (PLOD) 1 and PLOD3 lysyl hydroxylases (79). PLOD family proteins are strongly linked to multiple cancers, and several PLODs are recognized as a prognostic marker of gastric carcinoma (80). Future work will reveal whether these EBNA1-PLOD family interactions impact GC development and prognosis.

### 4.2 The functions of LMP1 and LMP2A in EBVaGC

Variable expression of both LMP1 and LMP2A can be detected in a subset of EBVaGC (70, 71). Both are transmembrane proteins located in the plasma membrane that function as constitutively active growth factor receptors. Together, in EBV infected B cells, they mimic normal antigen-derived signals that force differentiation into memory B cells, creating a long-lasting reservoir of EBV-infected cells and contributing to B cell oncogenesis (81, 82). The roles of LMP1 and LMP2A in EBVaGC are less clear, but are thought to similarly impact signal transduction pathways involved in epithelial cell growth and survival. Both have been recently reviewed in depth, although much remains to be discovered about their roles in EBVaGC (83, 84).

## 5 Role of EBV miRNAs in EBVaGC tumor progression

In addition to the two non-coding EBER viral RNAs expressed in all cells harbouring EBV, the EBV genome encodes up to 48 mature micro RNAs (miRNAs) (85). These viral miRNAs impact a variety of cellular functions, which include remodelling the cellular transcriptome, interference with immune signalling, and contribute to tumor progression and immune escape (86–88). Additionally, EBVs are known to produce long noncoding RNAs (lncRNAs), some of which play roles in hijacking human miRNAs and assisting viral replication, amongst other roles (89, 90). This section will give an overview of a number of miRNAs and lncRNAs produced by EBV and their association with EBVaGCs and other EBV-associated cancers.

### 5.1 The role of BART and BHRF1 miRNAs in EBVaGCs

**Figure 2:**
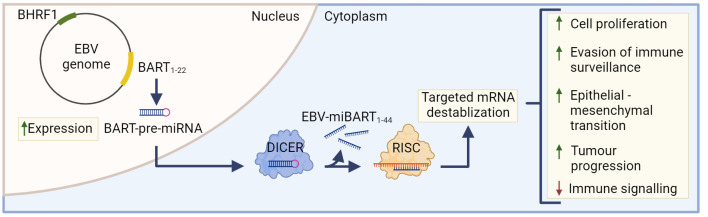
The role of EBV-miBARTS in EBV associated gastric cancer. The EBV genome encodes 22 BART-pre-miRNAs, which are highly expressed in EBVaGC. Following nuclear translocation, the BART pre-miRNAs are processed by Dicer, resulting in 44 EBV-miBARTs. EBV-miBARTs then guide the RISC complex to partially complementary sequences within target mRNAs, leading to targeted mRNA destabilization. This EBV-miBART-mediated regulation of gene expression promotes the expression of genes involved in tumor progression and survival, while decreases the expression of genes involved in immune signalling processes. Hypermethylation of the BHRF1 promotor leads to almost undetectable expression of BHRF1 miRNA in EBVaGC. Created with BioRender.com.

The most highly transcribed viral RNAs in EBVaGCs map within the BamHI-A region of the genome (22), including the BamH1-A rightward transcripts (BARTs) (**Figure 2**). These encode 44 intronic viral miRNAs from 22 precursor hairpins (91). The EBV-encoded BART miRNAs are a group of small regulatory RNAs under 100 nucleotides long and are often highly expressed in EBV malignancies (92), suggesting that they may play a role in tumorigenesis (93). Unlike most cellular miRNAs, both the 5’ and 3’ sides of the miR-BART precursor hairpins are efficiently loaded into the RNA-induced silencing complex (94, 95). Roughly 99% of all virally derived polyadenylated transcripts in EBVaGCs are from the 44 miR-BARTs (92, 95). The viral miR-BARTs are also highly expressed, representing >10% of the total pool of miRNAs in EBVaGCs (96).

These viral miRNAs regulate gene expression post-transcriptionally by guiding the RISC complex to partially complementary sequences within target mRNAs, leading to mRNA destabilization (**Figure 2**) (97). Although the current understanding of EBV miRNA function is far from complete, many viral miRNAs target viral and cellular factors involved in host cell growth, survival, signalling pathways, metabolism and anti-viral immune responses (91, 98–102). Much of the existing work has been done in lymphoid cells and the exact roles that most EBV miR-BARTs play during the processes of gastric carcinogenesis are virtually unknown (85). The only comprehensive experimental study to identify cellular mRNAs targeted by miR-BARTs utilized photoactivatable ribonucleoside-enhanced crosslinking and immunoprecipitation (PAR-CLIP) in latently infected lymphoblastoid cell lines. This identified 531 sites of interaction between seed sequences within EBV miRNAs and cellular 3’UTRs. This large number of interactions suggests that EBV miRNAs have profound and widespread effects on cellular gene expression (103).

Although in their infancy, existing studies of the roles of BART miRNAs in a variety of EBV malignancies have identified numerous functions, including the targeting of specific cellular transcripts (87), impairing NK cell-mediated recognition of infected cells (104, 105), decreased pathogen-recognition receptor (PRR)-mediated signalling and interferon induction (106), and impairment of antigen presentation pathways (101, 103).

Of note, EBV-miR-BART7-3p is one of the most highly expressed miRNAs in EBVaGCs (107) and plays a role in inducing cell proliferation and epithelial-to-mesenchymal transition (108). Additionally, EBV-miRNAs have been shown to modulate viral gene expression, often for the purposes of latent infections and evasion of immune surveillance (5, 109). Specifically, miR-BART5-5p and miR-BART19-5p are able to downregulate LMP1, an activator of many cellular immune signalling pathways (109, 110). Upregulated expression of EBV miRNA is associated with a 3-times higher mortality risk in NPCs and GCs (111), with miR-BART20-5p specifically associated with worse recurrence-free survival in EBVaGCs (112).

Although BamH1 fragment H rightward facing 1 (BHRF1) miRNAs are known to play a role in B cell transformation (113, 114), they are almost undetectable in GCs and NPCs (115). Indeed, the BHRF1 promoter is hypermethylated in almost all EBVaGC tissue, and this epigenetic modification likely contributes to its lack of expression (**Figure 2**) (116).

### 5.2 The role of lncRNAs in EBVaGCs

Long non-coding RNAs are defined as RNAs over 200 nucleotides long and lack protein-coding abilities (117). Both EBV and their host cells are known to produce a number of lncRNAs (89, 117, 118). In particular, several host cell lncRNAs are differentially expressed in EBVaGC, including small nucleolar RNA host gene 8 (SNHG8), which modulate the expression of a number of cellular and viral genes, as well induces cellular proliferation and invasion (119, 120). Though several EBV BART-encoded lncRNAs have shown to downregulate a number of genes *in vitro* (93), their *in vivo* functionality has not yet been confirmed.

## 6 Impact of genotype and polymorphisms on EBVaGC

Most EBV associated cancers, including NPC, Burkitt lymphoma and T/NK lymphomas, display distinct and non-overlapping geographic distribution patterns. In contrast, the frequency of EBVaGC shows no such regional variation (121). This suggests that regional environmental or host genetic differences may play a lesser role in EBVaGC compared to other EBV-associated cancers (121). It remains an open question whether genetic differences between EBV subtypes are associated with increased risk of developing EBVaGC post EBV infection.

There are two major genotypes of EBV, type 1 and 2, which differ in the sequence of a number of important viral genes (122). Type 1 EBV is the predominant strain in Western and Asian countries while type 2 EBV is frequently found in Africa. Significantly, EBV-1 and EBV-2 differ in their ability to transform B lymphocytes and epithelial cells into a state of continuous proliferation, as well as their association with both cancerous and non-cancerous disease (123, 124). Few studies have assessed if these genotypes, or the myriad of more subtle variants, contribute differently to the burden of EBVaGC (125). The possibility that functional differences between genetically distinct EBV variants contribute to CG risk clearly warrants further investigation on a large scale.

## 7 Hallmarks of cancer in EBVaGCs

All cancer features can be divided into “hallmark characteristics”, which include genomic instability, sustained proliferative signalling, replicative immortality, and angiogenesis (126, 127). Some of these hallmarks of cancer differ between EBVaGCs and EBVnGCs (34, 128–130). This section will explore the changes in the host genome, cell signalling pathways, and cell cycle regulation in EBVaGCs, and how such changes impact tumor progression and growth, as well as patient outcomes.

### 7.1 Genetic changes

**Figure 3:**
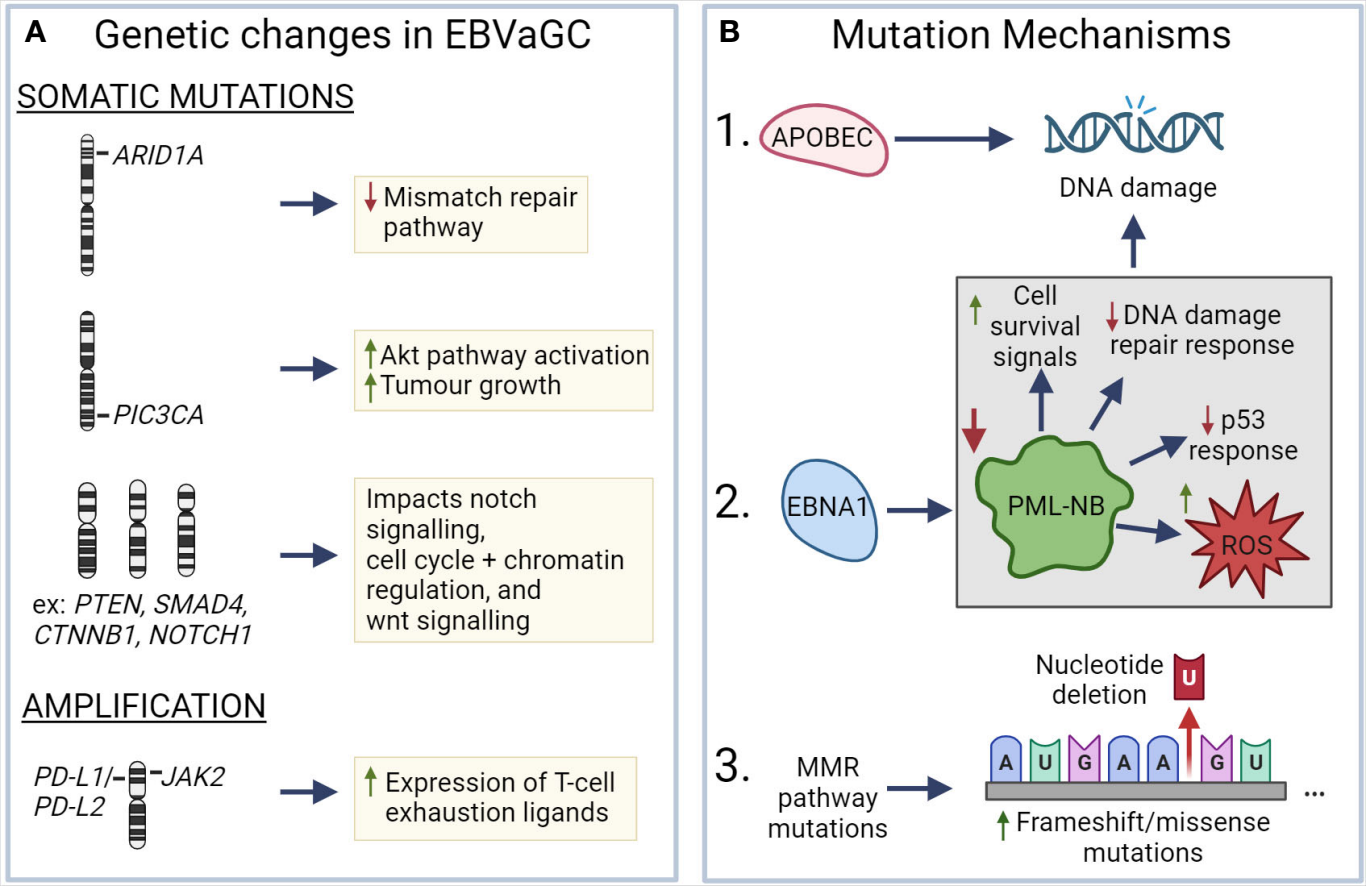
Known mechanisms that lead to genetic changes in EBV associated gastric cancer **(A)** *ARID1A*, *PIC3CA*, *PTEN*, *SMAD4*, *CTNNB1*, and *NOTCH1* exhibit frequent somatic mutations in EBVaGC, resulting in deficiencies in the mismatch repair pathway, increased AKT pathway activation, impacts on wnt and notch signalling, as well as cell cycle and chromatin regulation. EBVaGCs are also associated with amplification of the chromosomal regions encoding *JAK2, PD-L1* and *PD-L2*, which contributes to the increased expression of T cell exhaustion markers. **(B)** APOBEC and EBNA1, via downregulation of promyelocytic leukemia nuclear bodies (PML-NB), contributes to DNA damage. Frequent MMR pathway mutations result in an increase of frameshift and missense mutations. These different mechanisms can lead to genetic changes in EBVaGC. Created with BioRender.com.

EBV has been associated with a number of genetic changes within EBVaGCs (**Figure 3**). In particular, EBVaGCs are strongly associated with somatic mutations of PIK3CA and ARID1A, a feature it shares with the MSI GC subtype (131, 132). Mutations in PIK3CA are associated with increased activation of the Akt pathway, tumor growth, and invasiveness (133, 134), with the overexpression of PIK3CA being associated with poorer patient outcomes (**Figure 3A**) (135). Furthermore, PIK3CA can serve as a marker of tumor differentiation and morphology, with increased PIK3CA expression being associated with low grade tumor histology and intestinal-type GCs (135–137). Similarly, loss of ARID1A expression is associated with poorer outcomes in non-MSI EBVnGCs (138, 139), with decreased expression associated with TNM stage and depth of invasion (140) (**Figure 3A**). Furthermore, loss of ARID1A is also associated with the deficiency of the mismatch repair pathway and is correlated with the MSI subtype of GC (141, 142). Apart from PIK3CA and ARID1A, genes such as PTEN, SMAD4, CTNNB1, and NOTCH1 that are involved in Wnt and Notch signalling pathways, or cell cycle and chromatin regulation, are also frequently mutated in EBVaGCs (**Figure 3A**) (130). Interestingly, mutations of the tumor suppressor gene TP53 are rare in EBVaGC, albeit frequently mutated in other human cancers (143). Apart from mutations, EBVaGCs are also associated with amplification of 9p24.1, which contains the genes encoding JAK2, as well as PD-L1 and PD-L2, both of which are T cell exhaustion ligands (**Figure 3A**) (83, 130).

There are a number of mechanisms which may be responsible for the aforementioned genetic changes (**Figure 3B**). One such mechanism is APOBEC-mediated DNA damage in EBVaGCs, where it is associated with activating mutations in the PIK3CA kinase (144). EBNA1, an EBV-associated protein, is also associated with DNA damage via the downregulation of promyelocytic leukemia nuclear bodies, resulting in impaired DNA repair responses, reduced p53 responses, promotion of cell survival signals, and increased reactive oxygen species (ROS) production and accumulation (75, 145). Additionally, many of the DNA mismatch repair pathway genes are mutated in EBVaGCs (146), resulting in an increased number of frameshift and missense mutations in both oncogenes and tumor suppressor genes (147), though such a highly mutated phenotype is often associated with positive responses to immune checkpoint inhibitor therapy, as they can lead to the generation of tumor neoantigens (148–150).

### 7.2 Epigenetic changes

Apart from genetic changes, EBVaGCs often feature epigenetic dysregulation, whether through altered DNA methylation patterns, the expression of EBV-associated miRNAs, or variable regulation of human miRNAs. Based on the CpG-island methylator phenotype (CIMP), EBVaGCs belong to the high (CIMP-H) category (129, 151). In fact, over 1000 genes have been found to be differentially methylated by EBV, with hypermethylated genes enriched in Wnt and protein kinase signalling pathways, among other cancer-associated pathways (152–154). EBV is also noted to express a number of miRNAs, with said miRNAs functioning to inhibit pro-apoptotic genes and promote cell survival, as well as regulate the expression of EBV-associated genes (115, 155). Additionally, EBV is known to downregulate a number of human miRNAs with tumor suppressor functions, particularly members of the let-7 and miR-200 families (156).

## 8 Tumor microenvironment

The TME is the ecosystem of the tumor and surrounding regions, allowing the tumor to persist, expand, and spread metastatically (157). Due to the molecular and pathology-associated differences between EBVaGCs and EBVnGCs, variations within the TME are also expected and are of potential interest. In addition to cellular neoantigens derived from tumor-specific DNA alterations that give rise to novel protein sequences, EBVaGCs express foreign viral antigens that represent excellent targets for T cell responses (158, 159). The presence of these non-self viral antigens in EBVaGCs is likely a key factor influencing the overall anti-tumor immune response and altered tumor immune microenvironment as compared to EBVnGCs (160). This section will give an overview of the known differences present between EBVaGCs and EBVnGCs, as well as various aspects of the TME in GCs.

### 8.1 Immune infiltration and immune cell composition

EBVnGCs are considered to be “immune-cold” tumors, with little to no T cell infiltration (161–163). EBVaGCs, on the other hand, are “immune-hot”, with high infiltration of immune cells, including CD8+, CD4+, and dendritic cells (DCs) (164, 165). The high lymphocytic infiltration of EBVaGCs is a common defining histological characteristic compared to EBVnGCs (20). Indeed, in a recent cohort study of 421 primary GCs, the density of CD3+ T cells in the tumor epithelium was 3.27 times higher (P<0.0001) than the next highest GC subtype (166). In EBVaGCs, the ratio of CD8+ to CD4+ T cells is 10:1, indicating increased cytotoxic antitumoral activity (167), with RNA-seq analysis of GCs showing that cytotoxic CD8+ T cell (CTL) signatures are strongly correlated with EBV viral load, which is expected given that viral antigens will be recognized as non-self by the host immune system (168). Though the level of infiltrating DCs is higher in EBVaGCs as compared to EBVnGCs (43, 165, 167), few are CD1a+, resulting in tolerogenic responses of T cells against tumor cells (164) (169, 170). EBV-infected epithelial cells help facilitate this process by secreting exosomes that inhibit DC maturation and contributes to tumor progression (165). Additionally, the TME of EBVaGC promotes CD4+ differentiation to Foxp3+ regulatory T cells via PD-1/PD-L1 signalling, contributing to the immunosuppressive environment of the TME (171, 172). Genes related to Th17 differentiation are also differentially expressed in EBVaGCs (173), with accumulation of Th17 cells associated with tumor progression and metastases via IL-17 secretion (174), as well as poorer patient outcomes for GCs (175, 176). Furthermore, a unique subpopulation of biphenotypic B cells, expressing both B and T cell markers, have been previously identified in EBVaGCs, though their role has not been deciphered (177).

In addition to the beneficial immune cells within the TME, there are also cells actively contributing to the immunosuppressive TME. One such group of immunosuppressive cells are the tumor-associated macrophages (TAMs), which are most often associated with the anti-inflammatory M2 phenotype and contribute to tumor cell proliferation and angiogenesis (178, 179). EBVaGCs, in particular, have a greater proportion of pro-inflammatory M1 macrophages compared to EBVnGCs (180). Even so, the role of macrophages within EBVaGCs may depend on the proportion of M1/M2 macrophages, with M1 TAMs dominating at early stages of the tumor and M2 TAMs during later stages (181–183). Another group of immunosuppressive cells are myeloid-derived suppressor cells (MDSCs), which are an adverse prognostic factor in GCs (184, 185) and are known to inhibit adaptive and innate immune antitumoral responses via a variety of mechanisms (186). MDSCs arise from myeloid progenitor cells that have not terminally differentiated into mature granulocytes or macrophages. MDSCs are also associated with increased regulatory T cell presence within the tumor and the Th2 cytokine, IL-13 (185). Interestingly, Th2 skewing of the CD4+ T cell helper response makes it the predominant phenotype in GCs (187). However, there is conflicting information, with Th2 shown to display both anti-tumor activity and benefitting tumor growth (188, 189). Even so, higher ratios of Th1/Th2 cells are associated with better outcomes (190).

### 8.2 Differences in antigen presentation

Effective T cell-specific anti-tumor responses require the presentation of a tumor-associated antigens in either the context of major histocompatibility complex class-I (MHC-I) or class-II (MHC-II) (191). Surveilling antigen-presenting cells (APCs) acquire exogenous peptides and present them in the context of MHC-II on the APC cell surface to activate antigen-specific CD4+ helper T cells (192). In conjunction with the ligation of co-stimulatory molecules between the APC and T cell, this two step process triggers proliferation and survival of antigen specific T cells (193). These activated CD4+ helper T cells subsequently stimulate CD8+ cytotoxic T cells specific for the same peptide antigen. This CTL response targets and lyses tumor cells displaying that specific, endogenously-derived antigenic peptide in the context of cell surface MHC-I (194, 195).

A prevalent mechanism of tumor cell evasion of the CTL response is the loss or down-regulation of the presentation of tumor antigens by the cancer cell in the context of MHC-I (196). Intriguingly, EBVaGCs express higher levels of MHC-I than other GC subtypes (47, 197). Indeed, EBVaGCs display high mRNA levels for all MHC-I components, including heavy and light chains, as well as factors required for loading, in comparison to both normal control tissues or EBVnGCs (47). This is likely a consequence of higher IFN-γ levels in EBVaGCs (168, 198). Intratumoral IFN-γ is produced by tumor-infiltrating lymphocytes and is a known inducer of transcription of the genes encoding MHC-I and components of the antigen loading complex (199). Interestingly, EBVaGCs were recently reported as exhibiting the highest IFN-γ gene response signature of all the GC subtypes (200, 201). Thus, EBVaGCs may more effectively display endogenously-derived antigenic peptides compared to EBVnGCs, enhancing their detection and lysis by CTLs.

As mentioned above, presentation of viral or tumor-derived neoantigens occurs in the context of MHC-II molecules, which are primarily expressed by professional APCs, such as DCs, macrophages and B cells (202). However, exposure of epithelial cells to IFN-γ also induces expression of MHC-II. These epithelial cells can subsequently function as accessory APCs to present antigens and stimulate an effective CTL response (203). Increased levels of MHC-II proteins on epithelial cells should enhance the presentation of exogenously-derived viral- and tumor-specific peptide antigens, enhancing CTL responses (204). Indeed, the underappreciated role for tumor cell derived MHC-II in anti-tumor immunity is becoming apparent, with numerous reports suggesting that tumor-specific MHC-II expression is correlated with favorable outcomes in many cancer types, including GCs (205, 206).

Interestingly, EBVaGCs display high mRNA levels for virtually all MHC-II genes, as well as the MHC-II-like α- and β-chains, and the invariant chain encoded by CD74 as compared to EBVnGCs (48, 198). The coordinated upregulation of MHC-II pathway genes by IFN-γ is clearly illustrated by strong global correlations in transcript abundance (48). These conclusions are supported by single cell RNA sequencing (scRNA-seq) analysis that conclusively shows much higher expression of MHC-II mRNAs in malignant epithelial cells isolated from an EBVaGC case compared to EBV-negative GCs (207). Immunohistochemical analysis also confirms higher protein expression of various MHC-II classes in EBVaGCs (167, 207, 208).

The coordinated upregulation of the components of both the MHC-I and MHC-II antigen presentation pathways by higher IFN-γ levels, combined with the expression of exogenous viral antigens, may help explain why the clinical outcomes for EBVaGCs are superior to EBVnGCs. An analogous situation is present in the TME of human papillomavirus-associated head and neck cancers, which similarly display upregulated MHC-I and II pathway components and better patient outcomes compared to those without a viral etiology (209, 210).

### 8.3 Molecular mechanisms of immunosuppression

As compared to EBVnGCs, a number of immunosuppressive genes are upregulated in the TME of EBVaGCs. One such gene is *IDO1*, a potent inhibitor of immune cells, allowing virus-associated tumors to persist in opposition to the increased local immune cell concentration (164, 168). IDO1 functions by exhausting tryptophan within the TME, producing kynurenine in the process. Reduced environmental tryptophan results in reduced proliferation, immune cell function, and contributes to tumor escape and metastasis (211). Programmed cell death protein 1 (PD-1), encoded by *PDCD1*, and its ligand PD-L1, encoded by cluster of differentiation 274 (*CD274*), are also upregulated in EBVaGCs (53, 212). Typically, PD-1 is expressed on T cells, and PD-L1 on TAMs and tumor cells within the TME (213). The interaction of PD-1 and PD-L1 inhibits T cell proliferation and a number of effector functions (214, 215). Additionally, PD-L1 has been shown to play roles in modulating epithelial-to-mesenchymal transition and chemoresistance in many types of cancers, including GCs (216). A number of other immune checkpoint molecules are also expressed by activated immune cells in EBVaGCs. These include hepatitis A virus cellular receptor 2/Tcell immunoglobulin and mucin domain 3 (TIM-3), encoded by *HAVCR2*/*TIM-3* (217), lymphocyte activation gene 3 (LAG-3), encoded by *LAG3* (218), and cytotoxic T lymphocyte associated protein 4 (CTLA-4), encoded by *CTLA4* (219). These collectively modulate the magnitude and duration of immune response (220). The activation of these checkpoints has been noted in many other types of cancers and normally serves to protect the host from the negative consequences of sustained immune activation during infection or cancer (221). The overexpression of TIM-3, LAG-3, and CTLA-4 is also associated with non-response to immune checkpoint blockade monotherapy for PD-1/PD-L1 (222). Combined inhibition of immune checkpoints augments antitumor immunity and results in enhanced tumor clearance (223).

## 9 Immunotherapy and other treatments

Due to the differences in the TME and cellular targets present within EBVaGCs and EBVnGCs, there are also differences in overall treatment effectiveness and patient responses (40, 161, 164, 224). A number of therapies have been shown to be effective within EBVaGCs and related cancers, with this section highlighting those currently approved, those still undergoing testing, and potential immuno- and chemo-therapeutics (225–227).

### 9.1 Immunotherapeutics

EBVaGCs are known to exhibit differential gene expression compared to EBVnGCs, with some of these gene products serving as targets for immunotherapeutics. Both PD-1 and PD-L1 are overexpressed in EBVaGCs (53) and are associated with worse patient outcomes (228). Pembrolizumab, an anti-PD-1 antibody, is an immunotherapeutic approved for treating a wide range of cancers, with a favourable benefit-to-risk profile and impressive antitumoral activity (229, 230). In a ground-breaking study by Kim et al., an overall response rate of 100% was demonstrated in EBVaGCs, raising hopes that this therapeutic would revolutionize treatment for these cancers (231). It was also demonstrated that response-rate was related to PD-L1 expression, thus making EBVaGCs prime candidates for the testing of anti-PD-1 immunotherapeutic targets (232). Pembrolizumab is currently undergoing clinical trials for EBVaGCs (NCT04795661, NCT05166577). Camrelizumab, also an anti-PD-1 antibody, has shown inconclusive results, possibly due to low PD-L1 positivity and small sample size (233). Avelumab, another anti-PD-L1 antibody, has been considered for maintenance therapy for GCs as an alternative to first-line chemotherapeutic treatments, with it demonstrating increased safety, but no effect on overall survival (234, 235). Trastuzumab, an anti-HER2 antibody, has been tested in conjunction with chemotherapy, resulting in higher overall survival as compared to chemotherapy alone (236). The identification of a wider range of targets, as well as improved EBVaGC patient stratification likely to respond to these targeted agents, would increase effectiveness and diversity of options for monoclonal antibody therapy (237, 238).

Apart from antibodies, a number of other immunotherapeutic treatment options are being considered for treatment of EBVaGCs (239–241). One such option is chimeric antigen receptor (CAR) T cell therapy, with multiple clinical trials recruiting GC patients for these studies (NCT05583201, NCT05393986, NCT05396300, NCT04650451). Studies have shown that a number of antigens, which include HER2, CEA, EpCAM, Claudin 18.2, and NKG2D, could be potential targets for CAR T cell-based therapy (242, 243). CAR T cell therapy has been reported to provide 5 more incremental quality-adjusted life-years compared to 4.6 years for nonpharmaceutical approaches (surgery, radiation therapy, stem cell transplants), at a similar level of cost-effectiveness (244). However, CAR T cell therapy has a number of downsides, which include the side effects of targeting antigens present on tissues besides the tumor (245, 246), cytokine storm syndrome (247, 248) and decreased effectiveness due to the immunosuppressive environment often present within cancers (249). These issues can be partially or fully resolved with engineering CAR T cells to have inducible rather than constitutive cytokine production (250, 251) and employing CAR T cells in conjunction with immune checkpoint inhibitor therapy (252, 253). As an alternative to CAR T cell therapy, CAR natural killer cell (CAR NK-cell) therapy has shown promising results with human GC cell lines in mouse models (254). CAR NK-cell therapy appears to have several advantages, including a reduced risk of alloreactivity and graft-versus-host disease, and the ability to kill cancer cells through non-MHC-restrictive effects (255, 256).

Cancer vaccines represent another potential avenue for EBVaGC immunotherapy given the expression of “non-self”, virally encoded proteins, which should be highly antigenic. Such vaccines come in many flavors, including whole tumor cell vaccines, DNA vaccines, DC vaccines, peptide vaccines, cancer stem cell vaccines, and neoantigen-based vaccines (257). A number of these vaccines have been approved and been successfully employed in various cancers (258–261). In GCs and related cancers, a candidate vaccine against the gastrointestinal peptide gastrin inhibits tumor growth and metastases in addition to modifying the TME when given alone or alongside immune checkpoint inhibitors (262, 263). Currently, a DNA vaccine specific for EBVaGC and other EBV-associated cancers is currently undergoing preclinical trials (264). This vaccine targets the BARF1 antigen of EBV, which is highly expressed in EBVaGCs (265), and has been found to promote cell proliferation and block apoptosis (266, 267). BARF1 has previously been identified as a potential therapeutic target (268, 269). Results of the preclinical trial indicate that the vaccine elicits high titers of antibodies specific for BARF1 and supresses tumor growth via a CD8+ T cell mechanism (264).

The well-established ability of EBV to actively evade both intrinsic and adaptive immune responses via a plethora of different mechanism could compromise anti-tumor vaccines (5). Specifically, viral interference with the recognition of antigenic viral peptides via downregulation of MHC-I-based antigen loading and presentation during infection has been well described and could greatly blunt the effectiveness of MHC dependent T cell responses (5, 270). However, this may not be as significant of an impediment as anticipated, as recent studies have shown strong MHC-I and -II expression in EBVaGC, likely due to upregulation by the inflammatory cytokines in the TME (47, 48).

### 9.2 Chemotherapeutics

EBVaGCs are resistant to a number of chemotherapeutics, including 5-fluorouracil (271) and docetaxel (272), both of which are effective in EBVnGCs (273, 274). However, other chemotherapeutic options may effectively treat EBVaGCs. These include combination therapies, which exhibit improved effectiveness over monotherapies, with examples such as 5-fluorouracil with LY294002, a selective inhibitor of PI3K (271), the PI3K/mTOR dual inhibitor CMG002 with chloroquine (275), and ganciclovir with gemcitabine (276). Additionally, there is also the option of adjuvant therapy, in which surgical intervention is followed by a monotherapy or combination therapy. One such study employed gastrectomy followed by either capecitabine and oxaliplatin or S-1 monotherapy, although no difference in outcomes was observed between EBVaGCs and EBVnGCs (277). Additional studies were performed to identify better combinations of chemotherapeutics, although only limited results were achieved (278, 279).

Since EBVaGCs are often associated with hypermethylation of promoters within the genome, particularly those of tumor suppressors and negative regulators of the cell cycle (280, 281), drugs targeting DNA methylation using either DNA methyltransferase or histone deacetylase inhibitors have been suggested to have therapeutic potential. Currently, two DNA methyltransferase inhibitors, azacitidine and decitabine, have been approved for acute myeloid leukemia and myelodysplastic syndrome (282), but may also be employed in EBVaGCs. Another drug currently undergoing clinical trials, zebularine, was previously shown to demethylate the STING promoter, which regulates expression of the cGAS/STING innate immune pathway, resulting in increased CD8+ T and NK infiltration, as well as reduced tumor burden in GC cell line-based models (283). The benefits of these classes of drugs include the reversibility of effects, due to epigenetic changes, as well the possibility of reversing changes in the TME (154, 284). Downsides of such drugs include the potential of non-specific gene activation as a result of demethylation, which may include oncogenes, as well as the lack of studies exploring the long-term effects of epigenetic therapies (129). Further research in this class of therapeutics is necessary to determine their ultimate value as new treatment options for EBVaGC.

### 9.3 Drugs stimulating EBV reactivation from latency

EBV persistence in the infected individual is lifelong and largely mediated by the maintenance of the latent state of viral episomes in infected cells (285). Reactivation of latent virus could provide a means of killing EBVaGC cells, akin to the “shock and kill” strategies suggested for curing HIV (286, 287). In this way, reactivation of EBV infection from latency activates viral transcription, protein expression and virion production, potentially triggering cytolysis, immune-mediated clearance, and sensitivity to antiviral drugs such as ganciclovir and valganciclovir (288).

Reactivation of latent EBV infection is commonly referred to as lytic-induction therapy. This treatment approach typically involves a combination of a lytic inducer and nucleoside analogue antiviral pro-drugs (289). Reactivation of the EBV lytic cycle leads to the expression of numerous additional viral proteins not expressed during latency. These include the viral BGLF4 encoded protein kinase, which converts antiviral prodrugs like ganciclovir into their cytotoxic forms, consequently killing the infected cell (290) and adjacent cells via “bystander killing” (291). These additional viral products also potentially represent non-self antigens that may trigger improved anti-tumor responses or could be useful targets for CAR T cell-based therapy.

Multiple approaches for EBV lytic reactivation have been reported, primarily representing epigenetic-targeted therapies, such as histone deacetylase inhibitors (292, 293). Intriguingly, chemotherapy itself appears to trigger some level of EBV reactivation, which can be further enhanced by epigenetic modulators (294, 295). Although limited, existing studies assessing lytic induction therapy suggest potential utility in EBVaGC (276, 296), with ongoing screens searching for more effective small molecule inducers of EBV reactivation (297).

## 10 Datasets and tools in EBVaGC

This section serves to highlight public datasets and tools available for the exploration of EBVaGCs. The availability of said tools and datasets are most beneficial when performing *in vitro* or *in vivo* research, providing additional sources of validation and comparison. They can also assist in the process of hypothesis generation, by helping narrow down possible research targets that could be of interest when exploring oncogenesis, as well as potential biomarkers and therapeutic targets.

### 10.1 TCGA and TCGA-derived datasets

The Cancer Gene Atlas (TCGA) has a large collection of stomach adenocarcinoma (STAD) tissue samples, including bulk tumor gene expression data for up to 30 EBV-positive and 353 EBV-negative GC samples (22). The original dataset features patient clinical information, expression data for 20,531 cellular mRNAs and 1046 cellular miRNA genes, and methylation across 395,405 unique genomic probes. The aforementioned datasets can be accessed through the Broad Institute GDAC Firehose web portal (https://gdac.broadinstitute.org/) or other tools. Furthermore, additional sequencing of TCGA GC tissue samples have yielded datasets for expression levels of EBV-encoded mRNAs (201) and miRNAs (87), compilation of immune landscape features (298), and standardized patient outcome data (299). As one example of the utility of this data, bioinformatic comparisons confirmed that the EBVaGC TME exhibits many aspects of a T cell-inflamed phenotype, with greater T and NK cell infiltration, increased expression of many immune checkpoint markers (BTLA, CD96, CTLA4, LAG3, PD1, TIGIT and TIM3), and multiple T cell effector molecules in comparison with EBVnGCs (300).

### 10.2 EBVaGC-centric tools

The computational nature of working with molecular expression datasets may present a barrier to researchers not well-versed in computational techniques, which limits the accessibility of such datasets. While a number of viral-centric tools featuring molecular gene expression have been developed (301, 302), they are often few and far between. The same has been true for EBVaGCs up until recently; the development of the EBV-GCR web suite of tools (303) represents a useful addition. This tool set allows users to quickly query both viral and cellular gene expression data from single cell and bulk tumor GC mRNA sequencing data. Users can also easily obtain data regarding correlations between viral and cellular gene expression, genome-wide methylation, immune landscape features, and patient outcomes.

## 11 Perspectives and discussion

Even with the great wealth of existing knowledge related to EBVaGCs, there is still much to discover and understand to improve treatment options and patient outcomes. In particular, employing scRNA-seq for cancers can provide a deeper understanding of the TME composition, immune heterogeneity in tumors, evaluation of tumor escape mechanisms, as well as mechanisms of drug or therapy resistance (304). Currently, there are few studies leveraging scRNA-seq data for comparing EBVaGCs with EBVnGCs (207). Further, larger scale studies would be beneficial in understanding the various dimensions of EBVaGCs and their differences compared to EBVnGCs. Additionally, therapeutics targeting EBV-associated miRNAs are relatively unexplored (111) and given the roles for these virally encoded miRNAs in EBV-positive malignancies (100), could make for effective targets. An example of such a therapeutic was in a recent study in which researchers employed anti-miRNAs to silence miR-BART7-3p, reducing tumor growth in tested animal models (305). Another study found that circRNAome miRNA sponges of EBV can decrease the expression of multiple host miRNAs, thus helping drive carcinogenesis (306). In turn, microRNAs targeting EBV miRNAs could be developed in order to downregulate their expression. Advances in precision- and immuno-therapeutics should result in improved treatment options and patient outcomes.

## Author contributions

MS: Conceptualization, Writing – original draft, Writing – review & editing. KM: Conceptualization, Visualization, Writing – review & editing. JM: Conceptualization, Funding acquisition, Supervision, Writing – original draft, Writing – review & editing.
